# Long COVID and risk of erectile dysfunction in recovered patients from mild to moderate COVID-19

**DOI:** 10.1038/s41598-023-32211-5

**Published:** 2023-04-12

**Authors:** Hayder M. Al-kuraishy, Ali I. Al-Gareeb, Sumaiah J. Alarfaj, Rasha Khalifah Al-Akeel, Hani Faidah, Maisra M. El-Bouseary, Jean-Marc Sabatier, Michel De Waard, Thanaa A. El-Masry, Gaber El-Saber Batiha

**Affiliations:** 1 Department of Clinical Pharmacology and Medicine, College of Medicine, Al-Mustansiriyah University, Baghdad, Iraq; 2 Department of Pharmacy Practice, College of Pharmacy, Princess Nourah Bint Abdulrahman University, P.O.Box 84428, Riyadh, 11671 Saudi Arabia; 3 Department of Zoology, Faculty of Entomology and Parasitology, King Saud University, Riyadh, Saudi Arabia; 4 Microbiolgy Department Faculty of Medicine, Umm Al-Qura University, Mecca, Saudi Arabia; 5 Department of Pharmaceutical Microbiology, Faculty of Pharmacy, Tanta University, Tanta, Egypt; 6 CNRS UMR 7051, Faculté des Sciences Médicales et Paramédicales, Institut de Neurophysiopathologie (INP), Aix-Marseille Université, 27 Bd Jean Moulin, 13005 Marseille, France; 7 Smartox Biotechnology, 6 Rue Des Platanes, 38120 Saint-Egrève, France; 8 L’institut du Thorax, INSERM, CNRS, UNIV NANTES, 44007 Nantes, France; 9 LabEx «Ion Channels, Science & Therapeutics», Université de Nice Sophia-Antipolis, 06560 Valbonne, France; 10 Department of Pharmacology and Toxicology, Faculty of Pharmacy, Tanta University, Tanta, Egypt; 11 Department of Pharmacology and Therapeutics, Faculty of Veterinary Medicine, Damanhour University, Damanhour, 22511 AlBeheira Egypt

**Keywords:** Immunology, Microbiology, Endocrinology

## Abstract

Patients with coronavirus disease 2019 (COVID-19) were shown to have reduced serum testosterone levels compared to healthy individuals. Low testosterone levels are linked with the development of erectile dysfunction (ED). In this case-controlled study, 20 healthy controls and 39 patients with ED 3 months after recovering from mild-to-moderate COVID-19 pneumonia were studied. The patients ranged in age from 31 to 47 years. To identify early and late COVID-19 infections, real-time polymerase-chain reaction (RT-PCR) and COVID-19 antibody testing were done. The levels of luteinizing hormone (LH), follicular stimulating hormone (FSH), total testosterone (TT), free testosterone (FT), free androgenic index (FAI), and sex hormone-binding globulin (SHBG) were measured. The sexual health inventory for patients (SHIM) score was used to measure the erectile function of the patients and controls. When compared to the controls, the TT serum level in long COVID-19 (LC) patients with ED was low (*p* = 0.01). In contrast to controls, FT and FAI were both lower in LC patients with ED. (*p* = 0.001). FSH serum levels did not significantly differ (*p* = 0.07), but in ED patients, LH serum levels were elevated. SHIM scores were associated with low TT (*p* = 0.30), FT (*p* = 0.09), and high LH (*p* = 0.76) in LC patients with ED. Male patients with decreased serum levels of LH and testosterone may have hypothalamic-pituitary–gonadal axis dysfunction, which could lead to the development of LC-induced ED. Therefore, an in-depth research is necessary to confirm the causal link between COVID-19 and ED in LC patients.

## Introduction

**Figure 1:**
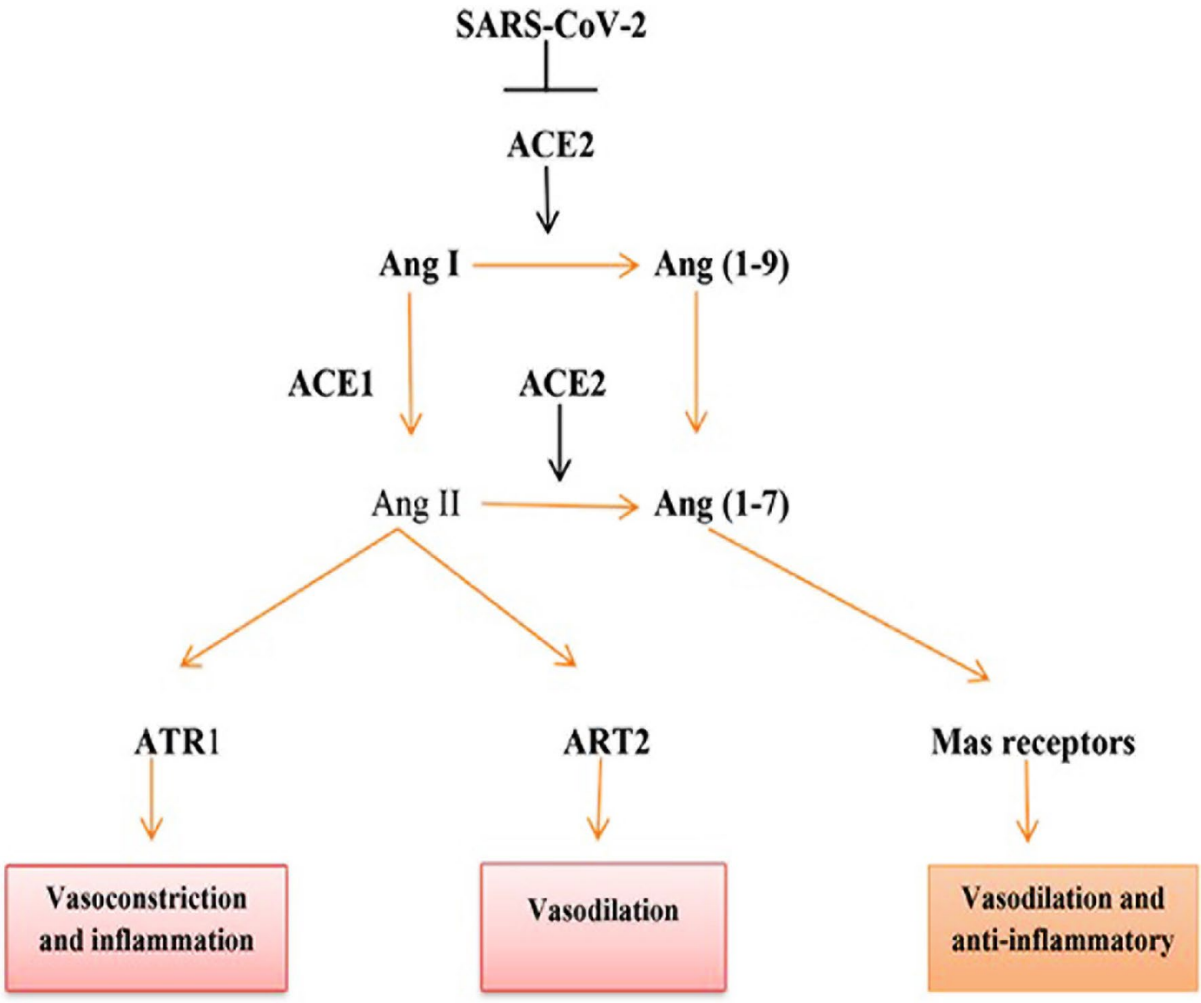
SARS-CoV-2 and disturbance of renin-angiotensin system: SARS-CoV-2-induced downregulation of ACE2 inhibits conversion of angiotensin I (AngI) to Ang-(1–9), and AngII to Ang-(1–7).

The world was affected by the most recent coronavirus pandemic in 2019 (COVID-19), which was brought on by the brand-new severe acute respiratory syndrome coronavirus 2 (SARS-CoV-2). SARS-CoV-2 is the largest member of the *betacoronaviridae* family and is an enveloped positive-sense RNA virus^1^. Angiotensin-converting enzyme 2 (ACE2), which is expressed in renal tubular cells, endothelial cells, lung alveolar cell type 2, cardiomyocytes, and testis, serves as both its receptor and entry point in the target cells^2^. Cellular transmembrane protease serine 2 (TMPRSS2) trims and activates the SARS-CoV-2 spike proteins to bind ACE2, which facilitates the binding of SARS-CoV-2 to ACE2^2^. Prostate, testis, and endothelium cells all have elevated levels of TMPRSS2 expression^3^. The renin-angiotensin system (RAS) is subsequently disrupted as a result of the interaction between the SARS-CoV-2 viruses and ACE2^3^. Angiotensin II (Ang II), a vasoconstrictor, is converted into the vasodilators Ang-(1–9) and Ang-(1–7) by the enzyme ACE2^4^. As a result, elevated levels of circulating Ang II cause endothelial dysfunction and enhanced pro-inflammatory cytokines, which are connected to ACE2 downregulation^5^ (Fig. 1).

Most COVID-19 patients recover with low mortality; however, some patients experience long-term symptoms, described as Long COVID (LC)^6^. LC was originally recognized as COVID-19 patients who had persistent symptoms for weeks after acute SARS-CoV-2 infection. These symptoms included dyspnea, fatigue, myalgia, insomnia, and cognitive and olfactory disorders^7^. However, these symptoms may last for months in some patients and negatively impact their daily activities^8^. LC can be classified as post-acute COVID-19 with symptoms that last longer than 3 weeks after the onset of the illness, or as chronic COVID-19 with symptoms that last more than 12 weeks^9^. However, previous studies have reported that patients with LC had persistent symptoms in varying frequencies and durations among COVID-19 survivor^10,11^.

Endocrine disruption in LC or testicular damage may be caused by hypothalamic-pituitary–gonadal (HPG) axis dysfunction brought on by SARS-CoV-2 infection^12^. A case–control study by Okcelik, 2021 found that compared to healthy controls, COVID-19 patients have lower testosterone serum levels^13^. Other research has shown how testosterone regulates human erectile function and male sexual responsiveness^14^. Additionally, erectile dysfunction (ED) has been connected to low testosterone levels^14^. A systematic review and meta-analysis of 204 published articles comprising 2092 COVID-19 patients and 1138 controls showed that SARS-CoV-2 infection is associated with a reduction of total testosterone (TT), and impaired sperm production^15^.

As an end, endothelial dysfunction, low serum TT levels, and SARS-CoV-2 infection and subsequent hypercytokinemia interact to cause ED in patients with LC. A SARS-CoV-2 infection may also result in long-term complications such cardiovascular problems, endothelial dysfunction, and endocrine dysfunction^9,12^. Therefore, early hyper-inflammatory status and associated multi-systemic injuries associated with acute SARS-CoV-2 infection may extend into COVID-19 survivors, causing LC and subsequently ED^8^. The present study hypothesizes that COVID-19 patients may experience long-term cardio metabolic, endocrine and neurological complications which affect the normal physiology of erectile function could result in ED^16^. Different studies revealed that LC patients were associated with development of ED^12,17^. However, the underlying mechanism for development of ED in LC patients was not fully elucidated. Therefore, this study aimed to elucidate the link between ED and testosterone in patients with LC.

## Materials and methods

The present case-controlled study involved 39 patients, aged 31–47 years, three months after overcoming mild-to-moderate COVID-19 pneumonia with the ED. They were recruited randomly from Al-Shiffa Medical Center, Baghdad, Iraq from June to October 2020, and were compared with matched 20 healthy controls that were recruited among relatives of the patients and outpatient visitors. The selection of patients with ED was performed according to the specific diagnostic criteria reported by Segraves, 2010^18^. The sample data were selected and drawn from a normally distributed population, and the sample size calculation was determined using baseline incidence, population variance, and treatment effect size with alpha = 0.05 and beta as power = 1 − β.

The Scientific Jury and Ethical Committee of Mustansiriyah University in Iraq granted permission and gave their seal of approval for the current study (ethical approval number 45QTR/2020) in accordance with the Helsinki Declaration. All participants provided knowledgeable consent for their participation in the study. Following detailed medical histories were collected and physical examinations, the control participants and selected patients were divided up into the following categories. Group A: LC patients with ED (*n* = 39), Group B: healthy controls (*n* = 20). The selection of patients with LC was accomplished according to established diagnostic criteria for LC^19^.

### Inclusion and exclusion criteria

#### Inclusion criteria

Patients who were male and had healed from COVID-19 but still had at least one lasting symptom, who were also diagnosed with ED (3 months after acute COVID-19 recovery), and were participating in this research and ranged in age from 31 to 47 years without previous history of ED prior COVID-19.

#### Exclusion criteria

The following patients were excluded from the study: those with a history of severe COVID-19, diabetes mellitus, hypothyroidism, congenital hypogonadism, chronic liver diseases, acute and chronic kidney diseases, acute and chronic bacterial infections, psychological and mental disorders, history of pelvic trauma, and those receiving treatment with diuretics or β-blockers.

### Anthropometric parameters

All participants had their systolic (SBP) and diastolic (DBP) blood pressures taken, and a specific equation was used to compute the pulse pressure (PP) and mean arterial pressure (MAP). PP = SBP-DBP, MAP = DBP + 1/3 PP^20^. Additionally, the explicit formula BMI = body weight (kg)/height (m^2^) was used to calculate body mass index (BMI)^21^.

### Biochemical parameters

All selected patients and controls had an overnight fast before having 10 mL of blood taken. The blood samples were centrifuged for 5 min at 3000 rpm that stored till time of analysis. Early and late SARS-CoV-2 infections were identified using real-time polymerase-chain reaction (RT-PCR) and COVID-19 antibody testing. For both IgG and IgM, the cut-off level was 0.00–0.04 mLU/mL, respectively.

Regarding hormonal assay, in this study, sex hormone-binding globulin (SHBG) and TT were evaluated using ELISA methods (Human testosterone and SHBG, ELISA Kit; MGC126834, Elabscience, Wuhan, China). Free testosterone (FT) was measured according to a previous study^22^. Free androgenic index (FAI) was calculated using the equation FAI = 100 × [TT/SHBG]^23^. Additionally, ELISA techniques were used to measure luteinizing hormone (LH) and follicular stimulating hormone (FSH).

### Erectile function assessment

LC patients and controls’ erectile functioning were calculated using Sexual Health Inventory for Men (SHIM) score^24^. SHIM which is a patient self-administrated questionnaire for assessment of male sexual dysfunction, scoring from 1 to 21 scores, with 98% sensitivity and 88% specificity^24^. The observed SHIM scores in the LC patients ranged from 1 to 21, with the following characterizations: normal =  > 21, mild ED is 17–21, moderate ED is 8–16, and severe ED is 1–7. The only observed scores in the LC/ED group ranged from 1 to 21. However, baseline psychiatric evaluations of the recruited patients were not performed.

### Statistical analysis

The mean ± standard deviation (*SD*), number (*n*), and percentages (%) used in this study were used to express and present the data. Data analysis was carried out utilizing SPSS, version 24. (IBM Corp., Armonk, NY, USA). To determine the significance of the difference between the LC patients and controls, an unpaired Student’s t-test was used. *p* values < 0.05 were regarded as significant.

### Ethics approval

The Scientific Jury and Ethical Committee of Mustansiriyah University in Iraq granted permission and gave their seal of approval for the current study (Ethical Approval Number 45QTR/2020) in accordance with the Helsinki Declaration. All individuals gave their informed consent to participate in the study.

### Consent to participate

All individuals taking part in the study gave their informed consent.

## Results

**Figure 2:**
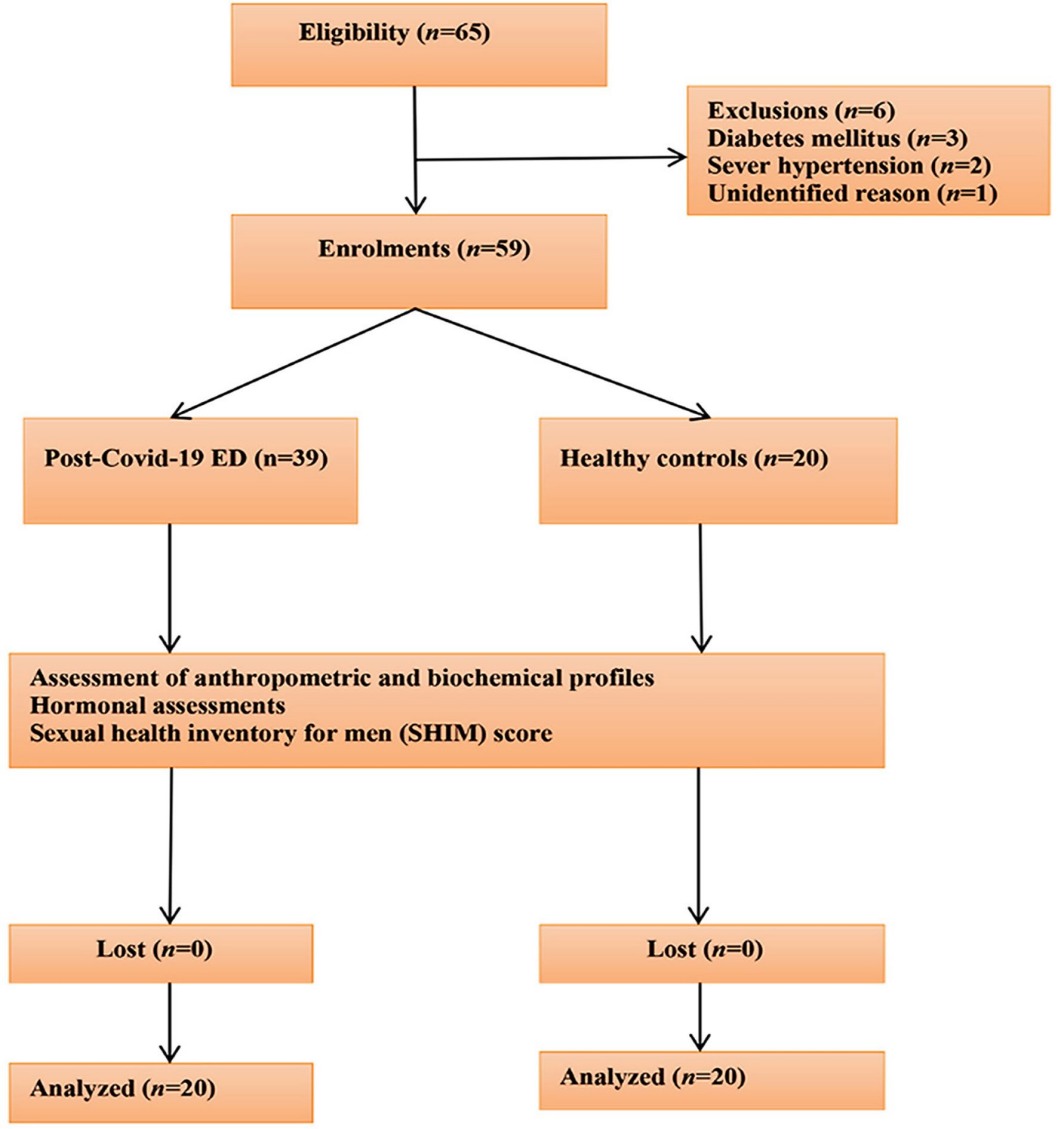
Consort-flow diagram for patients in the present study.

A total of 65 eligible patients and healthy controls were enrolled in the current study. Six patients were excluded due to diabetes mellitus (*n* = 3), severe hypertension (*n* = 2), and unidentified reasons (*n* = 1). Therefore, a total of *n* = 59 were recruited and continued the study, with *n* = 39 post-COVID-19 patients, and *n* = 20 healthy controls (Fig. 2). In the current investigation, LC patients with ED had blood pressure profiles that were higher than those of the controls (*p* < 0.01), with the exception of pulse pressure (PP) where the difference was not statistically significant (*p* = 0.06). Patients with LC have anti-SARS-CoV-2 IgG antibodies, and were associated with other comorbidities, including hypertension (23.07%) and asthma (7.69%). Regarding the patients’ response histories to phosphodiesterase inhibitors (PDEIs), most of the patients were responders, *n* = 30 (76.92%), compared to the non-responders (*p* = 0.04). Additional distinctive features are illustrated in Table 1.

**Table 1:** Cardio-metabolic and demographic features of post-COVID-19 patients with erectile dysfunction (ED) in contrast to controls.

Characteristics	Controls	Post-COVID-19 patients	*p*
*n*	20 (33.89%)	39 (66.10%)	0.01^#^
Age (years)	39.71 (7.08)	40.51 (7.33)	0.68
BMI = (kg)/(m^2^)	26.84 (3.22)	27 (3.19)	0.85
SBP (mmHg)	123.63 (6.28)	132.37 (9.54)	0.0005*
DBP (mmHg)	71.68 (5.95)	83.63 (6.12)	0.0001*
PP (mmHg)	51.95 (6.99)	48.74 (5.92)	0.06
MAP (mmHg)	89.00 (7.11)	99.88 (6.45)	0.0001*
Smoking	3 (15%)	4 (10.25)	0.69
Duration of ED (weeks)		8.21 (1.75)	
COVID-IgG (mIu/mL)	0.00	7.24 (1.32)	
Associated comorbidities			
Hypertension		9 (23.07%)	
Asthma		3 (7.69)	
Current pharmacotherapy			
Montelukast		3 (7.69)	
Theophylline		3 (7.69)	
CCBs		9 (23.07%)	
Respond to PDEIs			
Yes		30 (76.92%)	
No		9 (23.07%)	0.04

^#^*p* < 0.05, *p < 0.01.

*BMI* body mass index, *SBP* systolic blood pressure, *DBP* diastolic blood pressure, *PP* pulse pressure, *MAP* mean arterial pressure, *PDEIs* phosphodiesterase inhibitors.

**Figure 3:**
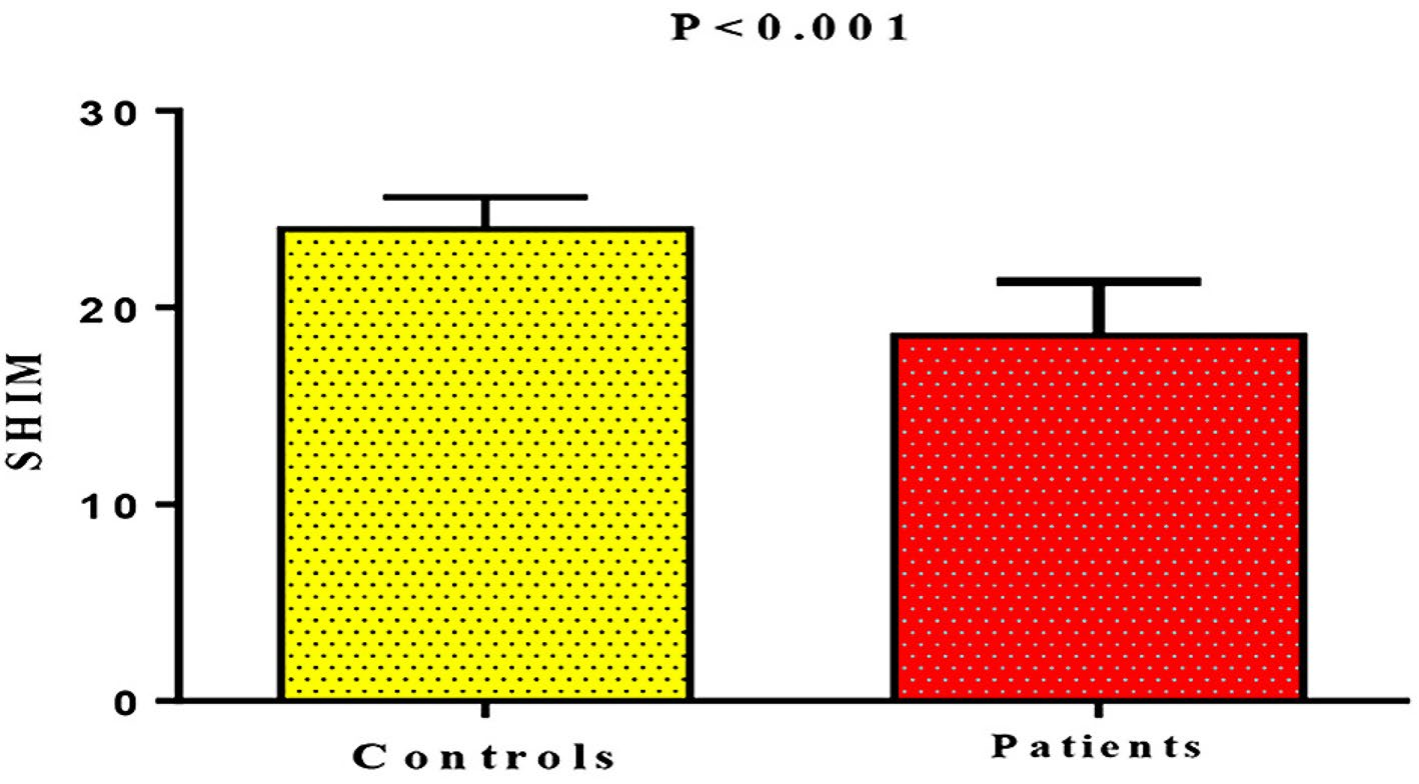
SHIM in post-COVID-19 patients with ED compared with controls.

Hormonal changes in LC patients with ED showed that TT serum levels were lower compared with the controls (18.52 ± 6.95 nmol/L vs. 22.95 ± 5.91 nmol/L, (*p* = 0.01). A similar trend was observed for FT and FAI (*p* = 0.001). FSH serum levels did not significantly differ (*p* = 0.07), although the LH serum levels were increased in LC patients with ED (Table 2). Regarding SHIM scores, LC patients with ED had lower SHIM scores compared with the controls (18.56 ± 2.75 vs. 23.98 ± 1.64, *p* < 0.0001) (Fig. 3). Moreover, SHIM scores were associated with low TT (*OR*, 2.18, 95% CI 0.48–9.87, *p* = 0.30), FT (*OR* 4.62, 95% CI 0.76–27.86, *p* = 0.09), and high LH (*OR* 1.33, 95% CI 0.20–8.70, *p* = 0.76) in LC patients with ED (Table 3).

**Table 2:** Hormone differences between erectile dysfunction patients and controls.

Parameters	Controls (mean ± *SD*)	Post-COVID-19 patients (mean ± *SD*)	95% CI	*p*
TT (nmol/L)	22.95 ± 5.91	18.52 ± 6.95	− 8.07 to 0.78	0.01^#^
FT (nmol/L)	0.27 ± 0.04	0.16 ± 0.07	− 0.14 to 0.076	0.001*
SHBG (nmol/L)	59.63 ± 1.89	53.75 ± 10.85	− 12.05 to 0.29	0.06
FAI	38.48 ± 6.06	34.45 ± 5.95	− 28.47 to 21.88	0.001*
FSH (mIu/mL)	3.04 ± 1.07	3.78 ± 1.64	− 0.07 to 1.55	0.07
LH (mIu/mL)	6.94 ± 1.78	6.11 ± 1.31	− 1.64 to 0.013	0.04^#^

^#^*p* < 0.05, *p < 0.01.

*TT* total testosterone, *FT* free testosterone, *SHBG* sex hormone-binding globulin, *FAI* free androgenic index, *FSH* follicular stimulating hormone, *LH* luteinizing hormone.

**Table 3:** Association of sexual health inventory for men in post-COVID-19 patients with erectile dysfunction.

Variables	*OR*	95% CI	*z* statistic	*p*
TT (nmol/L)	2.18	0.48–9.87	0.01	0.30
FT (nmol/L)	4.62	0.76–27.86	1.67	0.09
SHBG (nmol/L)	0.83	0.22–3.13	0.27	0.78
FAI	2.05	0.22–15.76	0.69	0.48
FSH (mIu/mL)	0.37	0.07–1.90	1.18	0.23
LH (mIu/mL)	1.33	0.20–8.70	0.30	0.76

*TT* total testosterone, *FT* free testosterone, *SHBG* sex hormone-binding globulin, *FAI* free androgenic index, *FSH* follicular stimulating hormone, *LH* luteinizing hormone.

## Discussion

Acute COVID-19 can certainly cause ED due to cytokine storm, endothelial dysfunction, cardiovascular instability, psychological impact, and hypoxia, as well as due to fear of sexual contact^25,26^. This novel study demonstrated the potential link between COVID-19 and ED in individuals who had recovered from mild-to-moderate COVID-19. Here, LC patients with ED were observed to have higher blood pressure profiles compared with healthy controls, with 23% patients being hypertensive and regular users of antihypertensive medications. Nonetheless, and based on previous studies, long-term cardiovascular complications are also linked to SARS-CoV-2 infection^27,28^. Additionally, LC may exacerbate underlying hypertension and other cardiovascular complications due to immunological and inflammatory instability^29,30^. It is noteworthy that the effects of LC appear to be very similar to other systemic diseases, such as diabetes mellitus, which cause microvascular dysfunction and autonomic neuropathy^31,32^.

In the present study, patients with ED have low TT and FT serum levels, and high LH serum levels compared with controls. Okcelik, 2021 confirmed that COVID-19 was associated with low TT serum levels due to suspected testicular injury induced by SARS-CoV-2^13^. It has been shown that Leydig cells and seminiferous tubule damage due to severe SARS-CoV-2 infection may impede spermatogenesis^33^. Therefore, the net effect of testicular injury in COVID-19 may adversely impact the androgenic profile and sex drive in patients with LC. It has been reported that testosterone is the primary regulator of sex desire, motivation, and penile erection in both humans and animals, thus, a reduction in testosterone may lead to the development of ED^34^.

Of note, thrombosis, cytokine storm, and hyperinflammation, SARS-CoV-2 infection may impair the HPG axis^35,36^. This may extend for a period and cause ED and male infertility. Due to a decrease in the production of LH and FSH from the anterior pituitary gland and gonadotropin-releasing factor (GnRF) from the hypothalamus, these pathological alterations may potentially result in central hypogonadism^37^. In the current investigation, LC patients' LH serum levels were lower than those of the controls, indicating dysfunction of the HPG axis. However, FSH serum level was insignificantly higher compared with the controls, which could be due to Leydig and Sertoli cell dysfunction^38^. Tabar et al. shown how the ACE2-mediated pathway may be used by SARS-CoV-2 infection to harm Sertoli cells and trigger the onset of male infertility^39^. Moreover, other endocrinopathies in LC patients, such as thyroid dysfunction and insulin resistance, may cause functional hypogonadotropic hypogonadism^12,40^. These findings suggest that dysfunction of the HPG axis in LC patients could be the possible mechanism for the development of ED.

Notably, down-regulation of ACE2 receptors in the testis and penile cavernous tissue leads to a reduction in testosterone secretion from Leydig cells^41^, and induction of AngII dependent-cavernous vasoconstriction due to reduction of Ang1-7/Mas bioactivity^42^. This is because testicular Ang1-7/Mas complex, which is expressed in Leydig and Sertoli cells, are distorted by high local or circulating AngII^43^. Thus, low testosterone-dependent ED could be caused by RAS dysregulation in COVID-19^44^.

Since high testosterone serum levels increase expressions of the angiotensin-converting enzyme-2/serine transmembrane protease serine 2 (ACE2/TRMPSS2) axes, which is essential for the entry and pathogenesis of SARS-CoV-2, it has been demonstrated that a reduction in testosterone levels in COVID-19 and LC patients may be a counter-balancing protective mechanism against SARS-CoV-2 propagation and induced complications^45^. Additionally, high testosterone serum levels increase the permissive effect of AngII through the expression of angiotensin type 1 receptors^46^. Similar to this, experimental study investigation from Mishra et al., 2016 showed that testosterone injection decreases the expression of the vasodilator angiotensin type 2 receptors (AT2R) in rats^47^. According to past and present experiences, testosterone therapy for the treatment of hypogonadism and ED in LC patients should be avoided because hyperandrogenic status is linked to the severity of COVID-19^48^. Of note, animal and human studies have reported that hypogonadism is linked with pro-inflammatory cytokines which may adversely affect erectile function mainly in aging men because testosterone blocks a variety of cytokines, including TNF-α, IL-1β, and IL-6^49^. Therefore, down-regulation of ACE2 by pro-inflammatory cytokines in hypogonadism may augment the seriousness of COVID-19 in men because of the negative correlation between COVID-19 mortality and the expression of ACE2^50^. However, testosterone exerts dimorphic roles in COVID-19 infections. At optimal levels, it is protective against the severe form for males but otherwise for females. It has been reported that testosterone serum level is reduced by aging and cardio-metabolic diseases including T2DM, obesity, dyslipidemia, heart failure, and atherosclerosis, which are common risk factors for the development of COVID-19 severity^51^. In addition, Pro-inflammatory cytokines, mainly IL-6, are involved in the pathogenesis of acute lung injury (ALI), acute respiratory distress syndrome (ARDS), and cytokine storm-induced multi-organ damage in COVID-19^52^. Of note, testosterone inhibits the synthesis and release of IL-6 and downregulates the expression of IL-6 receptors^51^. Therefore, IL-6 serum level is augmented in hypogonadism patients that increase their susceptibility for COVID-19 severity^53^. Therefore, COVID-19-induced reduction in circulating testosterone may induce ALI due to increase of pro-inflammatory.

Lower SHIM scores were also observed in patients with ED in comparison with the controls. These scores were associated with low TT, FT, FAI, and high LH, as reported by Cappelleri and Rosen, 2005^54^. Therefore, ED in LC patients has a complicated etiology and not simply attributable to low testosterone serum levels, as endothelial dysfunction, oxidative stress, pro-inflammatory cytokine hyper-activation, and high AngII serum levels are also interrelated in the onset of ED^55,56^. Furthermore, the response to the effect of phosphodiesterase type 5 inhibitors during sexual excitation could be a possible diagnostic tool for the assessment of ED^57^.

Notably, the autonomic nervous system is crucial for the regulation of male erection and ejaculation^58^, and autonomic dysfunction is associated with the development of ED^59^. Interestingly, different studies have revealed that LC patients experience dysautonomic features because auto-antibodies against muscarinic receptors and β adrenoceptors have developed^60,61^. Unfortunately, in the present study, autonomic functions were not assessed precisely. However, the blood pressure of LC patients was higher compared to the controls that might due to dysregulation of RAS by SARS-CoV-2 infection with increasing of pro-inflammatory and vasoconstrictor AngII, and reduction of anti-inflammatory and vasodilator Ang1-7^3,62,63^. Chen et al.^64^ from a cohort study confirmed that dysregulation of RAS in acute SARS-CoV-2 infection with increasing AngII could continue for long time and predispose for development of hypertension in LC patients. These verdicts proposed development of autonomic dysfunction in LC patients.

There underlying mechanism of LC-induced ED could be related to the development long-lasting endothelial dysfunction, autonomic neuropathy, endocrine dysfunction, testicular injury, and neuropsychiatric disorders^12,65^. However, the precise cause of ED in LC patients was not fully elucidated that needs further studies.

The present case-controlled study has a variety of limitations, including a limited sample size and a case-controlled study design that may affect the identification of causality and outcomes. Additionally, biomarkers of endothelial and oxidative stress and the ACE2/TRMPSS2 axis were not evaluated. Evaluation of anxiety, depression, stress levels, and psychological profile of the LC patients were also not conducted, although these factors may all interfere with erectile function. Furthermore, the overnight erection test, injection test, penile colored Doppler test, and ultrasound for ED that is more specific were not performed in the present study. Inflammatory biomarkers were also not estimated although they may reflect inflammatory status in LC patients. Given that ED, regardless of covid-19, increases with age, particularly after the age of 40, though we not have considered stratifying by age. In particular, LC patients with ED were not compared with LC patients without ED that is an integral point to show effect of LC on the hormonal changes and their relation in the development of ED. Nonetheless, despite of these limitations, strength of this case-controlled study it was regarded as an initial step toward authenticating the underlying causes of ED in LC patients. The present study confirmed that LC patients may experience a rate of ED due to reduction of testosterone levels.

## Conclusions

LC-induced ED may develop due to dysfunction of the HPA axis in male patients, as evidenced by reduced testosterone and LH serum levels. Therefore, in-depth research is required to confirm the causal link between COVID-19 and ED in LC patients.

### Clinical significance

The COVID-19 pandemic is brought on by SARS-CoV-2. There have been claims that COVID-19 patients have lower testosterone serum levels when compared with healthy controls. Additionally, low testosterone levels are linked with ED, so testosterone therapy may improve sexual function and prevent or reduce ED.

## Data Availability

All data generated or analyzed during this study are included in this published article.
